# Risk Factors, Pathophysiologic Mechanisms, and Potential Treatment Strategies of Futile Recanalization after Endovascular Therapy in Acute Ischemic Stroke

**DOI:** 10.14336/AD.2023.0321-1

**Published:** 2023-12-01

**Authors:** Gang Deng, Yun-hui Chu, Jun Xiao, Ke Shang, Luo-Qi Zhou, Chuan Qin, Dai-Shi Tian

**Affiliations:** Department of Neurology, Tongji Hospital, Tongji Medical College, Huazhong University of Science and Technology, Wuhan 430030, China

**Keywords:** acute ischemic stroke, endovascular therapy, futile recanalization, mechanism

## Abstract

Endovascular therapy is the first-line treatment for acute ischemic stroke. However, studies have shown that, even with the timely opening of occluded blood vessels, nearly half of all patients treated with endovascular therapy for acute ischemic stroke still have poor functional recovery, a phenomenon called “futile recanalization.”. The pathophysiology of futile recanalization is complex and may include tissue no-reflow (microcirculation reperfusion failure despite recanalization of the occluded large artery), early arterial reocclusion (reocclusion of the recanalized artery 24-48 hours post endovascular therapy), poor collateral circulation, hemorrhagic transformation (cerebral bleeding following primary ischemic stroke), impaired cerebrovascular autoregulation, and large hypoperfusion volume. Therapeutic strategies targeting these mechanisms have been attempted in preclinical research; however, translation to the bedside remains to be explored. This review summarizes the risk factors, pathophysiological mechanisms, and targeted therapy strategies of futile recanalization, focusing on the mechanisms and targeted therapy strategies of no-reflow to deepen the understanding of this phenomenon and provide new translational research ideas and potential intervention targets for improving the efficacy of endovascular therapy for acute ischemic stroke.

## 1. Introduction

Acute ischemic stroke (AIS) accounts for the vast majority of all strokes [1] and is one of the major causes of adult morbidity and mortality worldwide. Approximately 46% of AIS cases are due to acute intracranial large vessel occlusion (LVO) [2], which is the most devastating type of AIS. Endovascular therapy (EVT), especially mechanical thrombectomy (MT), with or without intravenous thrombolysis (recombinant tissue plasminogen activator, rt-PA) has been recommended as the standard treatment since the publication of five randomized controlled trials (RCTs) assessing the efficacy of MT in acute anterior circulation LVO in 2015 [3-7]. The efficacy of MT in basilar artery occlusion has also been validated recently in two RCTs conducted in China [8, 9]. However, about half of the patients who successfully underwent recanalization with MT did not achieve functional independence, a phenomenon termed futile recanalization (FR), both in acute anterior and posterior circulation occlusion [10-12]. Several clinical and imaging factors may independently predict FR and help in MT decision-making [13, 14], and FR may be partly attenuated by improved patient selection, shortened onset to recanalization time, reduced MT attempts, and refined postprocedural management [14, 15]. However, overcoming FR remains challenging as MT devices and techniques are updated and recanalization rates are increasing. Nowadays, reperfusion treatment combined with neuroprotection has attracted much interest and could be a promising approach for alleviating FR. As such, understanding the pathophysiology of FR is crucial and can help in searching for key intervention targets for FR.

## 2. Definition and occurrence rate of FR

FR commonly refers to a phenomenon in which patients do not achieve a sufficient long-term improvement in functional outcome (i.e., modified Rankin Scale [mRS] score of 3-6 or 4-6 at 3 months for patients with premorbid mRS ≤2), despite having successfully achieved occluded artery recanalization (grade 2b-3 on the Thrombolysis in Cerebral Infarction [TICI] scale or modified TICI [mTICI] scale in conventional angiography) [14].

The occurrence rate of FR was 43% in the HERMES study [10] which pooled individual patient-level data from five RCTs and ranged from 41 to 43% in late window MT studies [11, 12]. Few studies have assessed FR in posterior circulation. The only RCT reporting FR in the posterior circulation is the BEST study (FR 46%) [16]. A retrospective observational study found a higher rate of FR in basilar artery occlusion (47%) than in anterior circulation (data not reported in the article) [17].

Predictors of FR generally include advanced age, more severe stroke (higher NIHSS score and ASPECT), delayed MT treatment, cerebral small vessel disease (including leukoaraiosis and brain atrophy), and a high number of MT attempts [13, 14]. Our recent meta-analysis showed that several factors, such as female sex, pre-procedural comorbidities (hypertension and diabetes), higher systolic blood pressure and serum glucose, were associated with an increased risk of FR [14]. Other factors, such as collateral status and interleukin-6 and ADAMTS 13 level, may have predictive value for FR but further investigation is needed [14].

## 3. Mechanisms of FR and potential targeted therapy

**Figure 1.**
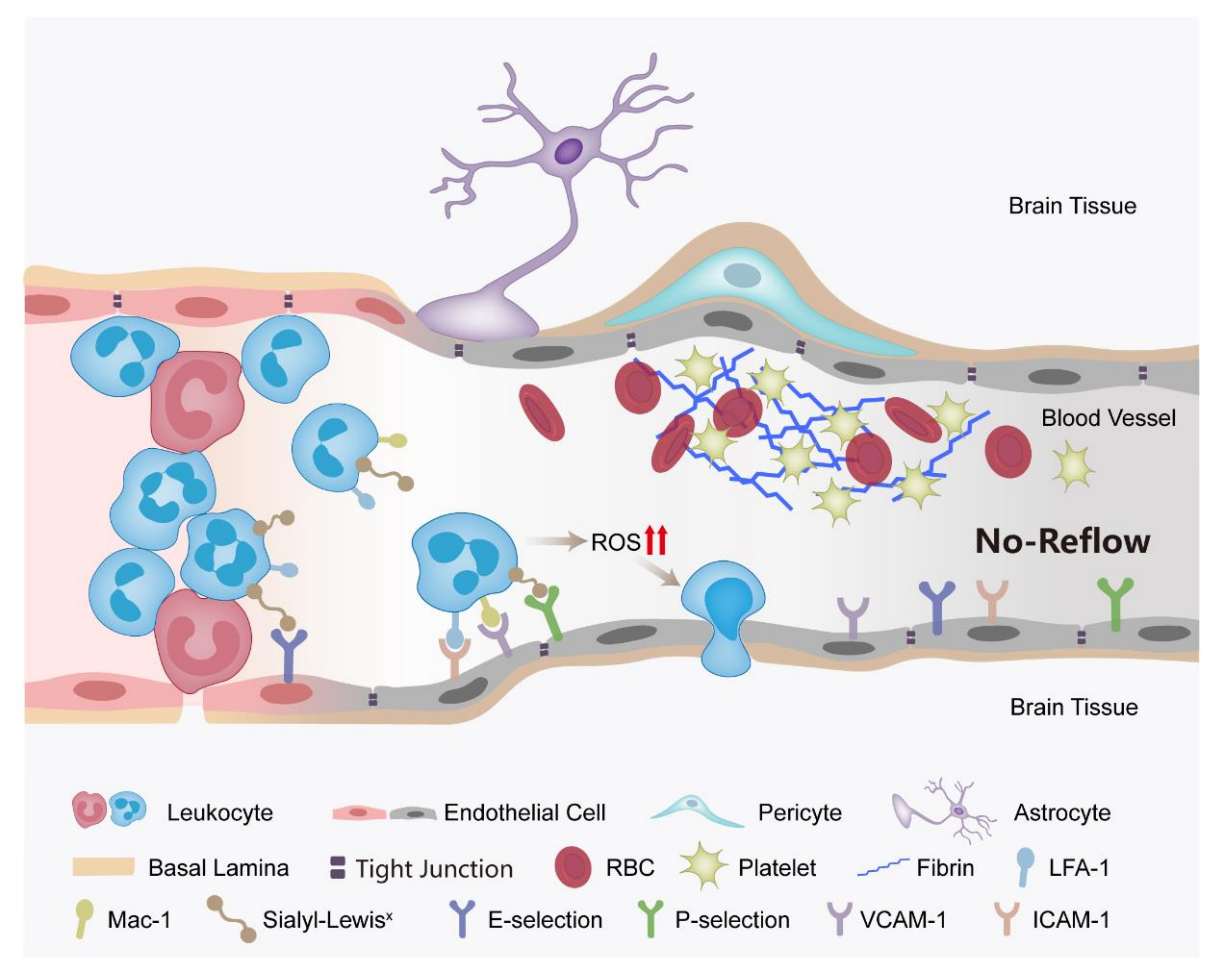
**Mechanisms of brain no-reflow phenomenon**. The mechanisms of brain no-reflow phenomenon involve adhesive molecule-dependent leukocyte-endothelial interaction and leukocytes clogging, aggregation of red blood cells and platelets and microthrombosis, swelling of endothelial cells and astrocyte end-feet, and contraction of pericytes.

**Figure 3.**
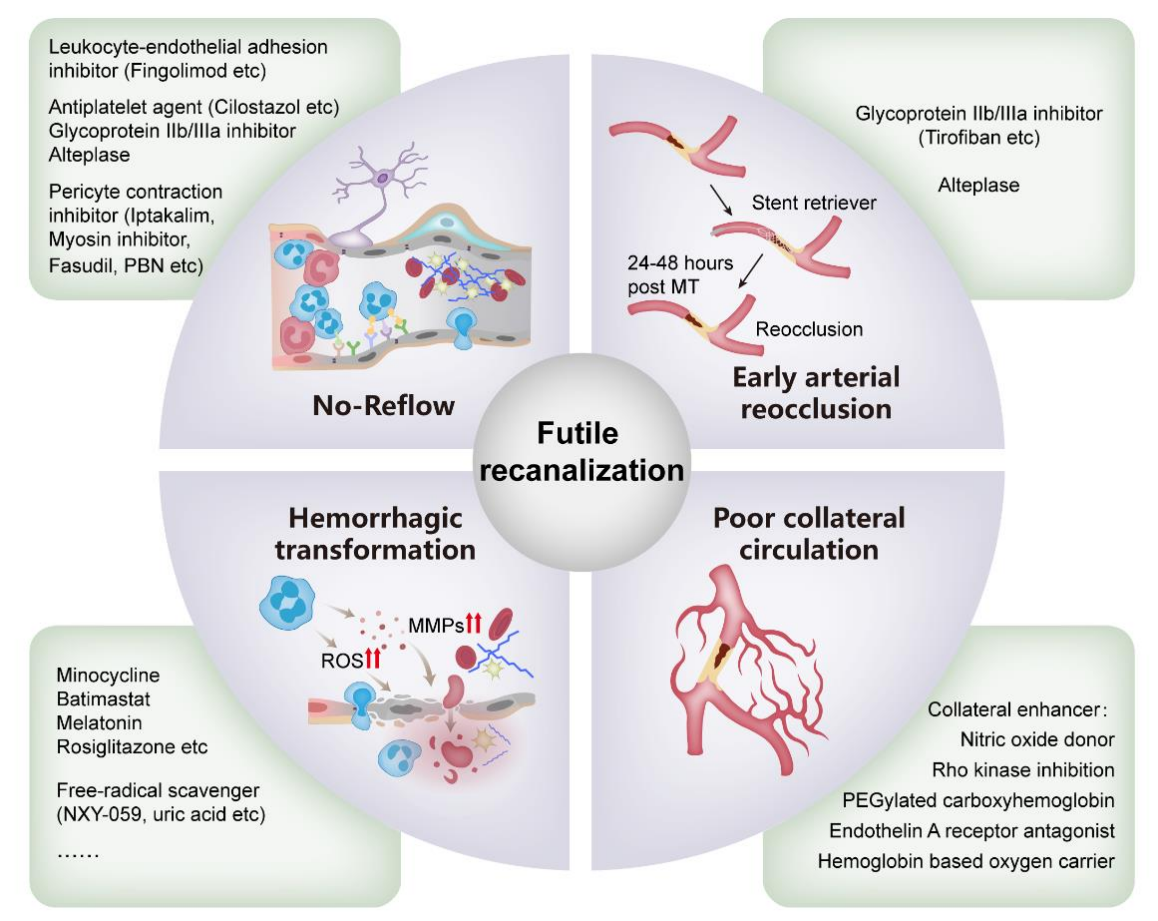
Potential mechanisms and corresponding therapeutic interventions for futile recanalization.

The pathophysiological mechanisms of FR are complex and unclear. The present review summarizes the potential mechanisms (Table 1, Fig. 1-3) and corresponding therapeutic interventions (Table 1).

**Table 1:** Therapies targeting the related pathophysiology of futile recanalization (FR) in acute ischemic stroke (AIS).

Drug/Therapy	Mechanisms of action	Targeting pathophysiology of FR	Application	Descripsion
**Anti-Ly6G antibody^77^**	Neutrophil Depletion	no-reflow	Thrombin-model of MCA stroke, mice	Neutrophil-depleting antibody
**Anti-E-selectin antibody^52^**	Inhibit LEA	no-reflow	tMCAO, mice	-
**Anti-P-selectin antibody^53^**	Inhibit LEA	no-reflow	tMCAO, mice	-
**anti-CD18 monoclonal antibody IB4^54^**	Inhibit LEA	no-reflow	tMCAO, baboon	-
**Genetic depletion of ICAM-1^55^**	Inhibit LEA	no-reflow	tMCAO, mice	-
Fingolimod^79,139,140^	Inhibit ICAM-1 expression and lymphocyte infiltration	no-reflow	tMCAO, mice	modulator of S1P receptor
		**HT**	tMCAO, mice/AIS patients	
**TP9201^82^**	Inhibit platelet adhesion and aggregation	no-reflow	tMCAO, baboon	Glycoprotein IIb/IIIa inhibitor
Cilostazol^66^	Inhibit adhesion molecules expression, platelet aggregation, and leukocyte plugging	no-reflow	tMCAO, mice	phosphodiesterase inhibitor
	**Inhibit MMP-9**	HT		
t-PA^83,86^	microvascular thrombosis	no-reflow	tMCAO, rat	-
		**EAR**	AIS patients	
Taurine^84^	Inhibit microvascular thrombosis	no-reflow	eMCAO, rat	-
	**Inhibit CD147-dependent MMP-9**	HT		
AS605240^85^	Inhibit microvascular thrombosis	no-reflow	eMCAO, rat	PI3Kγ inhibitor
		**HT**		
**PBN, L-NA, L-NIO^42^**	Inhibit ROS-induced pericyte contraction	no-reflow	tMCAO, mice	superoxide scavenger/NOS inhibitor
**Iptakalim^88^**	Inhibit pericyte contraction	no-reflow	tMCAO, mice	ATP-sensitive potassium channel opener
Fasudil^89,135^	Inhibit pericyte-induced vasoconstriction	no-reflow	Transgenic mice	Rho kinase inhibitor
	**Inhibit MMP-9**	HT	tMCAO, mice	
**Tirofiban^100,111^**	Inhibit thrombosis	EAR	AIS patients	Glycoprotein IIb/IIIa inhibitor
**Argatroban^113^**	Inhibit thrombosis	EAR	AIS patients	thrombin inhibitor
**NXY-059^126,128^**	Inhibit ROS	HT	eMCAO, mice/AIS patients	free radical scavenger
**Uric acid^127,129^**	Inhibit ROS	HT	eMCAO, rat/ AIS patients	free radical scavenger
**Edarvone^130,131^**	Inhibit ROS	HT	AIS patients	free radical scavenger
**Minocycline^132,133^**	Inhibit MMP-9 and inflammation	HT	eMCAO, rat/ AIS patients	broad-spectrum antibiotic
**Batimastat (BB-94)^134^**	Inhibit MMP-9	HT	eMCAO, rabbit	board spectrum MMP inhibitor
**Progesterone^135^**	Inhibit MMP-9	HT	tMCAO, rat	-
**Melatonin^135^**	Inhibit MMP-9	HT	tMCAO, mice	-
**Statins^135^**	Inhibit MMP-9 and module inflammation	HT	eMCAO, rat	-
**Rosiglitazone^135^**	Inhibit MMP-9	HT	tMCAO, rat	Sulfonylurea receptor 1 inhibitor
**Vinblastine^136^**	Depletion of PMN	HT	tMCAO, rat	non-specific neutrophil-depleting agent
**mAbRP3^136^**	Depletion of PMN	HT	tMCAO, rat	monoclonal anti-neutrophil antibody
**Tacrolimus^141^**	Inhibit inflammation	HT	MCA photothrombosis, rat	immunosuppressant
**Anti-VEGF antibody^135^**	Inhibit MMP-9	HT	eMCAO, rat	-
**PJ34^135^**	Prevent the degradation of TJPs	HT	eMCAO, mice	poly ADP ribose polymerase inhibitor
**HBO or NBO^135^**	Inhibit MMP-9	HT	tMCAO, rat	-
**hypothermia^135^**	Reduce metabolic state and the generation of ROS, inhibit MMP-2/9, suppress inflammation	HT	tMCAO, rat/AIS patients	-

LEA, leukoctye-endothelial adhesion; MCA, middle cerebral artery; tMCAO, transient MCA occlusion; ICAM-1, intercellular adhesion molecule-1; HT, hemorrhagic transformation; S1P, sphingosine-1-phosphate; MMP, matrix metalloproteinase; t-PA, tissue plasminogen activator; EAR, early arterial reocclusion; eMCAO, embolic MCA occlusion; ROS, reactive oxidative species; PMN, polymorphonuclear neutrophil; ADP, adenosine diphosphate; HBO, hyperbaric oxygen; NBO, normabaric oxygen.

### 3.1 No-reflow

#### 3.1.1 Evidence of brain no-reflow in preclinical and clinical settings

In experimental ischemia-reperfusion (I/R) models and patients with LVO who underwent recanalization, restoration of blood supply to large vessels does not necessarily result in reperfusion of microcirculation and ischemic tissue, leading to impaired capillary reperfusion, a phenomenon termed no-reflow, which has been described in many organs including the brain, heart, skin, kidneys and muscles [18]. Ames *et al.* first observed this phenomenon in a rabbit model of cerebral I/R using a cuff about the neck to prevent blood flow of the bilateral common carotid artery and basilar artery in 1960s [19]. The authors also found that the size of the no-reflow area increased with longer duration of ischemia and the blood components may have contributed to this phenomenon. Later experiments showed that no-reflow can be seen in a variety of animal brain I/R injury [20-22].

Clinically, the concept of no-reflow has gained much attention in the era of reperfusion therapy because reperfusion has emerged as a more precise predictor of outcome than recanalization [23]. No-reflow is associated with greater infarct growth and worse outcome [24]. The observation of no-reflow phenomenon in patients with clinical stroke just started three decades ago. In the first report investigating clinical cerebral no-reflow [25], after partial or complete recanalization with intra-arterial streptokinase determined by digital subtraction angiography (DSA), the perfusion deficit, assessed by single-photon emission computed tomography (SPECT), at 24 h of treatment was 25%. Later, several studies described no-reflow in 21-81% of patients with stroke who acquired partial or complete recanalization with intravenous alteplase [26]. However, partial recanalization *per se* may account for the reported hypoperfusions [26]. In addition, these studies have had major limitations, such as not using DSA to confirm recanalization, and the possibility of missing occlusion of the distal branches [26].

**Table 2:** Summary of studies on cerebral hypoperfusion (no-reflow) following successful recanalization of acute anterior circulation large vessel occlusion treated by endovascular therapy.

First Author (year)	Design	Time from onset to groin puncture	Time from onset to recanalization	Definition of Successful recanalization	Time to assessing reperfusion	Method assessing reperfusion	Operational definition of no-reflow	FR rate
**Marks 2014^27^**	Prospective	6.0 (4.7~8.3) h	NR	TICI 2b-3	Within 12 h	PWI	≤50% reduction in the PWI lesion which had a Tmax of >6 s comparing with the baseline PWI	6/40 (15%)
**Ng 2018^28^**	Retrospective	193 (154~301) min	NR	TICI 2b-3	1~3 d	TCD	MCA PI >1.2 or RI >0.75, or >20% comparing with the contralateral side	27.9%
**Rubiera 2020^29^**	Prospective	NR	287 (177~492) min	mTICI 3	30 min	CTP	Tmax >6 s	40/94 (42.5%)
**Schiphorst 2021^26^**	Retrospective	150 (124~202) min	196 (154~230) min	mTICI 2c-3	24 (20~27) h	ASL	≥40% reduction of CBF in the affected ROI relative to mirror ROI + infarction of the affected ROI on follow-up MRI	1/33 (3%)
**Ng 2022^24^**	Retrospective	NR	255 (200~290) min	eTICI 2c-3	24 h	CTP+MRP	persistent hypoperfusion on relative CBV or CBF maps within the infarct and verified quantitatively by >15% asymmetry compared to a mirror homologue in the absence of carotid stenosis or re-occlusion	33/130 (25.3%)

FR, futile recanalization; NR, not reported; TICI, Thrombolysis in Cerebral Infarction scale; PWI, perfusion weighed imaging; Tmax, time to peak of the residue function; TCD, Transcranial Doppler; MCA, middle cerebral artery; PI, pulsatility index; RI, resistance index; mTICI, modified Thrombolysis in Cerebral Infarction scale; CTP, computed tomography perfusion; ASL, arterial spin labeling; CBF, cerebral blood flow; ROI, region of interest; MRI, magnetic resonance imaging; eTICI, extended Thrombolysis in Cerebral Infarction scale; MRP, MRI dynamic susceptibility contrast-enhanced Perfusion Imaging; CBV, cerebral blood volume.

Few studies have evaluated the no-reflow phenomenon in patients with acute LVO treated with MT [24, 26-29] because the applicability of this approach has been established in only the last 8 years (Table 2). No-reflow indicates perfusion deficit post successful MT, which can be assessed with DSA at the end of MT, but there were inconsistencies in defining “successful” MT: TICI 2b-3, mTICI 2c-3, mTICI 3, or extended TICI (eTICI) 2c-3 were suggested in different studies (Table 2). Perfusion deficit can be verified by computed tomography perfusion (CTP), magnetic resonance perfusion weighed imaging (PWI), arterial spin labeling (ASL), or SPECT, of which CTP, PWI, and ASL may be preferred because of easier accessibility and lower cost. The occurrence of no-reflow varied considerably from study to study, ranging from 3 to 42.5%, which may be attributed to different devices, scales and timing assessing recanalization and reperfusion and different cut-off points for hypoperfusion. Different modalities may differ in sensitivity and specificity, as CTP and PWI may be contaminated by contrast staining, and ASL may be less sensitive. Besides, recanalization status affects no-reflow; patients with partial recanalization (mTICI 2b) at the macrovascular level are more likely to have perfusion deficits than those with complete recanalization (mTICI c) [29]. Finally, each study had a unique operational definition of perfusion deficit and no-reflow. It is notable that Schiphorst *et al.* observed a significantly lower rate of no-reflow (3%) because they used a considerably higher threshold for assessing perfusion deficit (≥40% of regional cerebral blood flow [CBF] reduction) [26]. Accurate identification of patients with perfusion deficits is of great importance as it may lead to adjuvant neuroprotective therapy, which has not been successful until now.

#### 3.1.2 Role of neurovascular unit (NVU) in no-reflow

The unique characteristic of no-reflow in the brain that differs from no-reflow in the heart and other organs is mainly due to the special structure of the brain-blood interface, the NVU, which consists of neurons, gliocytes (microglia, astrocytes, and oligodendroglia), and vascular cells (endothelial cells, smooth muscular cells, pericytes and basal lamina matrix) [30]. Endothelial cells, basal lamina matrix, astrocyte end-feet and pericytes form a specialized blood-brain barrier (BBB). The endothelial glycocalyx, namely the glycoproteins and proteoglycans covering the luminal surface of vascular endothelial cells, also constitutes part of the NVU and has an impact on CBF [31]. The NVU is critical for neurovascular coupling, CBF regulation, and brain perfusion pressure maintenance under physiological and pathological conditions [30]. After cerebral ischemia, the altered structure and function of the NVU contributes to no-reflow.

In a global cerebral ischemia model, an early pathological study suggested that the narrowed capillary lumen resulting from endothelial blebs, swollen endothelial cells and perivascular glia was responsible for the no-reflow phenomenon, instead of intravascular clotting or platelet thrombi [32]. These findings have also been observed in focal cerebral ischemia models [33-36]. Garcia *et al.* demonstrated that the swelling of astrocytes and endothelial cells promptly began within an hour after reperfusion [35]. However, Fischer *et al.* did not find severe pericapillary glial swelling or bleb formation and suggested that other mechanisms were involved [37]. Pericapillary cells swelling alone is not sufficient to result in incomplete microcirculatory reperfusion [38], as mathematical models suggests that it is also affected by reperfusion and blood osmotic pressure, and microvessel permeability [39]. In fact, the increased permeability of the BBB post I/R allows fluid extravasation and leads to interstitial edema, or even erythrocyte extravasation (hemorrhage), thus increasing the interstitial pressure and compressing microvessels [39, 40], which contribute to no-reflow. A pooled analysis of the EXTENDIA TNK and EXTEND-IA TNK part 2 trials showed that cerebral edema was associated with CBF reduction in patients with successful recanalization [24], which supports the role of vasogenic edema in no-reflow.

Pericytes have been presumed to be contractile cells and capable of regulating the size of the microvessel lumen and CBF, which was previously assumed to be modulated by vascular smooth muscular cells of precapillary arterioles. In an ex vivo study, Peppiatt *et al.* demonstrated that simulated ischemia could evoke pericytes contraction and reduce capillary diameter in the retina and cerebellar slices [41]. Yemisci *et al.* provided the first in vivo evidence of pericyte contraction during cerebral ischemia [42]. They found that the prolonged contraction of pericytes, despite the reopening of the occluded artery, was induced by oxygen and nitrogen free radicals released from the microvasculature, leading to capillary constriction, obstruction of erythrocyte flow, and reduced tissue survival, which could be reversed by nitric oxide synthase inhibitors [42]. Hall *et al.* demonstrated that cerebral ischemia induced sustained pericyte contraction and subsequent pericyte death, which may irreversibly compress capillaries and damage the BBB [43]. During reperfusion, pericyte marker alpha-smooth muscle actin expression increased with pericyte contraction, and positively correlated with reperfusion time [44]. Multiple signaling pathways may lead to pericyte contraction and death after stroke apart from oxidative stress, including elevated intracellular calcium concentrations, ion pump dysfunction, impaired separation between myosin and actin due to lack of ATP molecules, and excitotoxicity [45].

#### 3.1.3 Role of blood components in no-reflow

In their first report about no-reflow, Ames *et al. *suggested that blood components may contribute to the no-reflow phenomenon, as there were no perfusion deficits when flushing the cerebrovasculature with Ringer solution before induction of ischemia [19]. Later, Fischer *et al.* demonstrated that hemodilution with saline greatly reduced the amount of postischemic vascular impairment and suggested that the main culprits of cerebral no-reflow were increased blood viscosity and erythrocyte aggregation [46].

Accumulation of polymorphonuclear leukocytes (PMN) in brain regions with hypoperfusion has been observed in a global cerebral ischemia model [47]. The role of PMN in cerebral no-reflow was first investigated by Grøgaard *et al.* [48]. They showed that anti-neutrophil serum treatment prior to the ischemic insult resulted in significant improvement of local CBF. An electron micrographic study verified PMN accumulation in capillaries obstructing blood flow following middle cerebral artery occlusion (MCAO) in baboons [49]. Leukocyte plugging in microvessels may result from reduced deformability and increased adhesiveness. Leukocytes in patients with AIS showed decreased deformability [50] and may mechanically occlude microvessels. Moreover, the rolling and adhesion of leukocytes to the endothelium, which is adhesion molecule-dependent, play a key role in leukocyte clogging [51]. When ischemia occurs, P-selectin is mobilized to the surface from storage granules in endothelial cells and this is paralleled by an upregulation of E-selectin [51]. P-selectin and E-selectin bind to leukocytes to promote the rolling process, leading to cell activation of leukocytes and resulting in conformational changes and increased adhesiveness of lymphocyte function-associated antigen-1(LFA-1), as well as mobilization of macrophage antigen-1 (Mac-1) [51]. β2 integrins (LFA-1 and Mac-1, both refer to CD18) mediated leukocyte adhesion likely involve an interaction with intercellular adhesion molecule-1(ICAM-1) and vascular cell adhesion molecule-1 on endothelial cells [51]. Treatment with adhesion molecule neutralizing antibodies [52-54], or genetic depletion of adhesion molecules [55-57] in animal I/R models improved capillary perfusion, suggesting a role of adhesion molecule-dependent leukocyte plugging in no-reflow. Post stroke inflammatory signals also lead to increased leukocyte rolling and adhesion along the vascular wall within postcapillary venules, decreasing blood flow in cerebral I/R [58]. In addition, the rolling and adhesion of leukocyte promotes fibrinogen deposition and thrombosis in post-capillary venules [59].

Platelet aggregation has been observed within the microvessels of ischemic regions [35, 49, 60-63]. Platelets can be activated through the microvasculature following I/R, leading to the formation of platelet-platelet and platelet-leukocyte aggregates [64]. Platelet depositions was significantly attenuated by the combination of heparin and ticlopidine administered before ischemic insult [62]. Inhibition of the glycoprotein IIb/IIIa receptors results in improved CBF and reduced infarct volume [65]. Cilostazol, an antiplatelet agent, prevented no-reflow in focal cerebral ischemia [66]. These findings support the role of platelets in no-reflow. Fibrin deposition, tissue factor, and stacked red blood cells have all been shown to participate in microcirculation no-reflow in AIS after intravenous thrombolysis [67-69]. The interactions among leukocytes, platelets, vessel walls and coagulation during cerebral I/R induce downstream microvascular thrombosis and BBB disruption [70]. In the clinical setting no-reflow may be potentially induced by thrombectomy due to thrombus fragmentation and micro-embolism of distal microvessels [71, 72].

The I/R-induced disruption of the endothelial glycocalyx may likely contribute to leukocyte-endothelial interaction-dependent capillary plugging [73]. The erosion of the glycocalyx leads to the exposure of the endothelial cells to adhesion molecules, promoting the rolling and adhesion of leukocytes as well as platelets [73]. However, the role of the glycocalyx in cerebral no-reflow remains largely unknown in contrast to coronary no-reflow.

#### 3.1.4 Role of reactive oxidative species (ROS) in no-reflow

Cerebral I/R induces the production of ROS, which can influence almost all of the abovementioned mechanisms. The sources of ROS include xanthine oxidase, NADPH oxidase, mitochondria, and uncoupled nitric oxide synthase (NOS). ROS can promote the rolling and adhesion of leukocytes to endothelial cells by inducing the generation of inflammatory mediators. Hydrogen peroxide has been shown to up-regulate β2 integrins in leukocytes [74]. Moreover, ROS can upregulate the expression of adhesion molecules (including selectins and ICAM-1) via NF-kappaB activation [51]. ROS can also damage endothelial cells, cause increased permeability in the BBB [75], and mediate capillary no-reflow by inducing pericyte contraction [42, 76].

#### 3.1.5 Potential strategies targeting no-reflow

At present, there is no clinically available therapy to combat no-reflow. Preclinical studies have demonstrated the efficacy of some approaches that target no-reflow. Leukocyte-endothelial interaction is the first choice of a therapeutic target. Animal experiments have shown that clearance of neutrophils using an anti-Ly6G antibody relieves microvascular hypoperfusion [54, 77, 78]. Inhibition of leukocyte adhesion by adhesion molecule antibodies, such as antibodies against P-selectin, E-selectin, and ICAM-1, also prevents no reflow after thrombolytic treatment with rt-PA in mice with cerebral ischemia [52, 53, 55]. Fingolimod, a modulator of the sphingosine-1-phosphate receptor, has been shown to directly inhibit endothelial ICAM-1 expression in stroke models, which may contribute to alleviating the no-reflow phenomenon [79]. The genetic depletion of adhesion molecules has also been shown to decrease no-reflow [53]. However, clinical trials have shown that pharmacologic inhibition of leukocytes is futile or detrimental in patients with AIS [80, 81].

Inhibition of platelet-fibrin interactions has been attempted in several studies to dissolve downstream microvascular thrombosis. Glycoprotein IIb/IIIa inhibitors can prevent microvascular occlusion after experimental MCAO but increase the occurrence of parenchymal hemorrhage [82]. Cilostazol, a phosphodiesterase inhibitor and a clinically used antiplatelet agent, prevents no-reflow in focal cerebral ischemia [66]. Desilles *et al.* showed that alteplase administered before reperfusion reduced downstream microvascular thrombosis and increased microvessel patency [83], which is in accordance with our meta-analysis, suggesting that intravenous thrombolysis (IVT) before MT was associated with less frequent FR [14]. Taurine and phosphoinositide 3-kinase-γ inhibitor AS605240 were both shown to reduce microvascular occlusion when combined with alteplase compared with alteplase alone [84, 85]. Recently, an interesting study, the Chemical Optimization of Cerebral Embolectomy (CHOICE) trial evaluated the efficacy of adjunct intra-arterial alteplase after successful recanalization with MT in patients with LVO-AIS and found that the use of adjunct intra-arterial alteplase was associated with a better outcome [86]. A nested advanced brain imaging study in the CHOICE trial revealed that compared to placebo, adjunct alteplase enhanced brain reperfusion following successful MT [87], which means that for the first time, an approach has succeeded in preventing brain no-reflow in a randomized controlled trial.

Pericytes are another potential target. ROS-induced pericyte contraction was suppressed by superoxide scavenger N-tert-butyl-α-phenylnitrone (PBN), a low dose of NOS inhibitor N^ω^-nitro-L-arginine or L-N5-(1-iminoethyl)-ornithine, which improved microcirculatory reperfusion, and provided neuroprotection [42]. 2-sulfo-phenyl-N-tert-butylnitrone, a BBB-impermeable analog of PBN, can also restore microcirculatory patency and perfusion after recanalization by reducing vascular wall ROS [76]. The ATP-sensitive potassium channel opener iptakalim can ameliorate microvascular disturbance by inhibiting pericyte contraction after ischemic stroke [88]. Optogenetic stimulation of pericyte-induced vasoconstriction and slow blood flow can be reversed by fasudil, a Rho kinase inhibitor, suggesting its potential in blocking no-reflow [89]. The myosin inhibitor blebbistatin can inhibit pericyte contraction and is a drug that potentially ameliorates no-reflow [90]. Other modulators of pericytes may involve ATP [91], adenosine [92], and lactic acid [93].

### 3.2 Early arterial reocclusion (EAR)

#### 3.2.1 Mechanisms of EAR post MT

In the literature, EAR is always defined as a recurring occlusion at the same site 24-48 h after initial angiographic complete or partial recanalization of an occluded cerebral artery [94-99]. Embolization in previously normal territories is generally not considered as reocclusion. The incidence of EAR ranges from 2.3 to 9% after successful recanalization with MT [94-99]. Although EAR is not common in clinical settings, it is significantly associated with increased early neurological deterioration, poorer functional outcome and increased mortality [94]. The risk factors for EAR after MT have not been fully established and may include advanced age, target occlusion at the intracranial internal carotid artery, atherosclerotic pathology, no pre-treatment statin or antiplatelet therapy, more complex MT procedure, residual thrombosis or stenosis, and high levels of platelet or D-dimer [94-99]. The incidence of EAR in intracranial atherosclerotic stenosis (ICAS) can be as high as 18.4% [100], which requires aggressive intervention.

Theoretically, the major mechanism of EAR is endothelial injury [101-103]. The vascular endothelium and atherosclerotic plaques can be easily injured during MT, which elicits platelet activation, adhesion and aggregation, and tissue factor exposure, and lead to activation of the clotting cascade [104]. An enhanced signal in the vessel wall was observed post MT on high-resolution magnetic resonance imaging, which represents endothelial injury at the site of reocclusion [102]. Autopsy studies on patients with underlying ICAS post MT also revealed vascular pathological abnormalities including disruption of the fibrous cap, intraplaque hemorrhage, and subintimal dissection, which presumably contribute to EAR of the recanalized artery [105, 106]. Rare preclinical studies have investigated the pathogenesis of reocclusion after I/R, highlighting the crucial role of endothelial injury, platelet adhesion, fibrinogen deposition, and activation of the clotting system [107, 108].

#### 3.2.2 Management of EAR and potential biological target

First, early identification of EAR is very important because timely rescue therapy may improve the outcome. Closer monitoring, including with Transcranial Doppler, should be applied in patients at high risk of EAR. The optimal endovascular rescue therapy for EAR remains unclear, and first-line therapies to achieve revascularization include repeated MT, balloon angioplasty, and/or stent angioplasty [100, 101, 109, 110]. Further comparative studies are warranted to determine the efficacy and safety of these rescue approaches.

Endothelial damage tends to be more severe as recanalization is delayed, thus increasing the frequency of reocclusion of the target vessel after MT. Therefore, optimizing the workflow of early stroke management and improving the thrombectomy apparatus and skills to shorten reperfusion time are of great importance to reduce arterial reocclusion. Long-term statins and antiplatelet therapies inhibit platelet aggregation and vascular inflammation, which may provide a protective effect on the vascular endothelium to prevent thrombosis after MT [97].

As EAR shares some of the common pathogenesis with no-reflow, such as the activation of platelets and coagulation cascade [100], drugs aimed at fighting against no-reflow by inhibiting platelet-fibrin(ogen) interaction may be helpful in reducing EAR. Two retrospective studies indicated that intra-arterial or intravenous infusion of glycoprotein IIb/IIIa inhibitor tirofiban was associated with reduced risk of EAR in patients with ICAS post MT [100, 111]. However, in a recently published trial RESCUE BT, no significant difference in reocclusion was observed between patients receiving intravenous infusion of tirofiban and those who did not [112]. This may be because this trial included patients with and without ICAS, and tirofiban was only effective in preventing EAR in patients with ICAS as the primary functional outcome suggested. Argatroban, a thrombin inhibitor, was shown to sustain blood flow and reduce reocclusion in a femoral arterial occlusion rabbit model [113], but it has never been tested in a brain I/R model. Adjunct alteplase post successful recanalization with MT may also decrease EAR occurrence but in the CHOICE trial, patients with reocclusion were excluded [86]. Given the low incidence of EAR in non-selected (with both ICAS and non-ICAS pathology) patients with LVO-AIS, a large sample size will be needed to design a RCT testing the efficacy of a drug on EAR in this population. Hence, it is better to design an RCT targeting EAR in patients with ICAS-related LVO-AIS.

### 3.3 Hemorrhagic transformation (HT)

HT refers to cerebral bleeding within an area of primary ischemic stroke. The incidence of HT ranges from 10 to 40% in patients with ischemic stroke and is associated with increased mortality and morbidity [114]. HT can be classified as symptomatic (sICH) or asymptomatic intracerebral hemorrhage (aICH), clinically, or hemorrhagic infarction (HI) and parenchymal hemorrhage (PH), radiologically, according to the European Cooperative Acute Stroke Study criteria [115]. HI was further sub-classified as HI-1 (small petechial hemorrhagic infarction) and HI-2 (confluent petechial hemorrhagic infarction), and PH was sub-classified as PH-1 (<30% of infarct, mild mass effect) and PH-2 (>30% of infarct, marked mass effect). HT can be a consequence of the natural progression of stroke or a serious complication of reperfusion therapy [116]. Although the SKIP trial showed that intravenous rt-PA increased the occurrence of HT and sICH in patients undergoing MT [117], a recent meta-analysis revealed a comparable occurrence of sICH between the two groups [118]. Our previous meta-analysis identified sICH as a risk factor for FR after EVT [14]. Recently, a case-control study suggested that HI-2, PH-1, and PH2 were independent predictors of poor outcomes for patients with AIS after successful recanalization with EVT [119]. Therefore, reducing HT, especially sICH or PH represents an important strategy to combat FR.

#### 3.3.1 Mechanisms of HT after MT

The disruption of the BBB plays a crucial part in the pathogenesis of HT after AIS [116, 120]. It involves multifactorial processes and factors that can interact with each other. The initial ischemia results in energy failure, ATP depletion and a decrease in Na-K ATPase activity, leading to BBB breakdown via a cascade of cellular and molecular events that involve leukocyte accumulation and production of MMPs (matrix metalloproteinase) and ROS [116]. MMPs can directly degrade tight junction proteins and extracellular matrix components [121]. ROS can disrupt the NVU through damage to endothelial and smooth muscle cells, pericytes, and astrocytes, leading to increased permeability of the BBB [121].

Reperfusion treatment induces a cascade of detrimental events that can increase the likelihood of HT [120]. Alteplase may promote HT via both thrombolytic and non-thrombolytic mechanisms. Reperfusion-induced ROS can disrupt NVU and lead to the infiltration of neutrophils, which have the capacity to secrete a large amount of MMP-9 [121]. Hence, neutrophil-derived MMP-9 plays a key role in reperfusion-induced HT [122]. MMP-2 also takes part in reperfusion-induced HT, as it mediates degradation of tight junction proteins, causing disruption of the BBB in the early stage of AIS [123]. In addition, alteplase may promote HT independently of thrombolysis-induced reperfusion. It enhances the release of MMP-9 from neutrophils, astrocytes, and neurons [124].

HT post MT also involves both reperfusion and non-reperfusion mechanisms. First, MT induces rapid and sudden reperfusion. Second, the MT maneuver will impose direct damage to the endothelium and disrupt the integrity of the BBB [125]. It is not surprising that HT is a major complication of MT.

#### 3.3.2 Potential strategies targeting HT

Preventing HT lies in the preservation of the BBB. ROS reduction and MMP inhibition in the acute phases are promising targets for BBB protection [116]. The free radical scavengers NXY-059 [126] and uric acid [127] have been shown to reduce alteplase-induced HT in animal stroke models, which, however, failed to be replicated in patients with AIS [128, 129]. The effect of edaravone, another free radical scavenger, in reducing HT after AIS in clinical settings remains conflicting [130, 131] and needs further investigation.

Early, but not delayed, MMP inhibition has been shown to reduce BBB permeability and the occurrence of HT [116]. Minocycline, a broad-spectrum antibiotic, inhibits MMP-9 and brain tissue inflammation and reduces HT in rats [132], but fails to translate to the bedside for HT prevention [133]. Batimastat (BB-94), a board-spectrum MMP inhibitor, decreases alteplase-related HT in rats [134]. Other agents that have been shown to reduce HT by inhibiting MMPs in preclinical studies include progesterone, melatonin, statins, and rosiglitazone [135].

Modulation of inflammation represents another important strategy to prevent HT. BBB disruption and the incidence of HT in rodent stroke can be attenuated by depleting leukocytes with vinblastine or anti-neutrophil antibody [136, 137], or by using a CD11b/CD18 antagonist to inhibit neutrophils [138]. Fingolimod reduced the degree of HT after I/R in mice [139] and in patients with AIS [140]. Tacrolimus can also attenuate HT in rats by inhibiting inflammatory responses [141].

Other approaches targeting HT include cilostazol, fasudil, VEGF inhibition, poly ADP ribose polymerase inhibitor PJ34, hyperbaric or normabaric oxygen, and hypothermia [135].

### 3.4 Poor collateral circulation

The brain’s collateral circulation is composed of arterial anastomotic pathways capable of supplying perfusion to brain regions where normal blood flow have become compromised, as occurs in AIS. A large body of evidence suggests that the collateral circulation plays a crucial role in the prognosis of AIS. In experimental ischemic models, good collateral status was associated with reduced induction of heat shock protein, decreased inhibition of protein synthesis, and less progression of ischemic tissue [142]. Conversely, poor collateral flow correlates with faster ischemic core progression and greater final infarct volume [143]. Moreover, collateral status can predict the response to EVT. Good collaterals were associated with increased rates of recanalization and reperfusion [144], higher rates of favorable functional outcomes, and reduced rates of sICH and mortality [145] after EVT. Mechanistically, collateral blood flow prevents impairment of vascular function, thereby improving reperfusion after recanalization [77]. However, the correlation between collateral circulation and FR is still controversial, and no study has explored the efficacy of strategies targeting the collateral circulation to improve FR after EVT.

Potential approaches to enhance collateral circulation (mainly leptomeningeal collateral) include endothelin A receptor antagonist, Rho kinase inhibition, nitric oxide donor, hemoglobin-based oxygen carrier and PEGylated carboxyhemoglobin, which have only been confirmed in preclinical research, with some evaluated in clinical trials [142].

## 4. Conclusions and future perspectives

The main management strategy for FR should consider the following three points. First, the population selection for EVT should be optimized. Many studies have explored FR predictors, and some have established predictive models. However, due to the limited treatment measures for AIS, the decision to use EVT tends to be more liberal in the real world than in RCTs, so currently predicting FR does not change clinical practice and outcomes. Second, the EVT workflow, devices and techniques, as well as postprocedural management, should be optimized. The "Time is brain" concept of the time window is still the primary factor limiting reperfusion treatment in the acute phase of stroke, and the time from the onset to reperfusion is also an independent risk factor for FR after EVT. Therefore, optimizing pre-hospital stroke management and improving the stroke green channel is still the top priority of AIS treatment. Similarly, improving EVT devices and techniques, including anesthesia methods, thus reducing the time to recanalization, the number of passes, and increasing the proportion of complete recanalization can all reduce the incidence of FR. The management of blood pressure and antithrombolic treatment post-EVT may have an important influence on FR, but the optimal target of blood pressure and antithrombolic strategy are currently uncertain and need further investigation [15]. Third, neuroprotective approaches, especially when administered in combination with EVT should be developed; this has been investigated in a large number of preclinical studies but failed in translational trials. This may be due to a lack of understanding of the basic pathophysiology of FR.

The pathogenic mechanisms underlying FR are complex and remain unclear. The correlation between HT and collaterals with FR is controversial, and no study has explored them as therapeutic targets for FR. Several studies have shown that EAR after EVT is relatively rare [96-98], while in ICAS-related LVO-AIS, the incidence of EAR is as high as 18.4% [100], suggesting that the main contributors to FR vary in different populations and need specific interventions accordingly. The no-reflow phenomenon may be the core mechanism of FR, but related research is still in the early stage, and mostly refers to studies exploring the coronary no-reflow phenomenon. However, cerebral microcirculation is quite different from the coronary microcirculation, and the NVU, as a specialized structure, has unique physiological and pathological responses. Moreover, the lack of ideal animal models mimicking no-reflow post EVT also limits relevant basic and translational investigations. Although the transient MCAO model can mimic the recanalization process of EVT, BBB disruption and penumbra, it is limited for simulation of human FR due to its non-thrombotic occlusion pattern and large variation of infarct volume [146]. However, neither the endothelin-1 stroke nor the photothrombosis models could mimic the rapid recanalization process of EVT [146]. Therefore, previous preclinical studies have hardly examined the role of no-reflow on the background of EVT instead of intravenous alteplase. There are also obstacles to clinical studies on no-reflow. Currently, there are no accepted devices, scales, or cut-off points for assessing hypoperfusion after recanalization.

In general, the abovementioned mechanisms of FR interconnect with each other. The no-reflow phenomenon and BBB disruption (HT as a severe type) are different aspects of microvascular responses to I/R [40]. Ng *et al.* demonstrated that BBB disruption was negatively correlated with CBF [147], which can be partially explained by the fact that BBB disruption-induced cerebral edema (or HT) imposes pressure on microvessels and contributes to no-reflow. They share some common pathogeneses, including ROS and inflammation. Supporting this, strategies targeting these two processes can both improve capillary perfusion and attenuate BBB disruption and HT, as we previously mentioned. No-reflow and EAR both involve endothelial injury, platelet activation and aggregation, and activation of the clotting system. Collaterals affect recanalization and reperfusion [144], as well as HT following EVT [145]. The enhancement of collaterals helps increase cerebral reperfusion and reduce HT [40]. In summary, targeting some of the common pathways involved in FR may be of great importance as they provide protection against multiple events in the I/R cascade. For example, combining antioxidants with anti-inflammatory drugs may be a promising future direction [148]. However, as different individuals may have a certain mechanism as the predominant cause of FR, it is crucial to distinguish, predict and manage them accordingly in clinical settings.

In addition to the abovementioned mechanisms, other mechanisms, such as excitotoxicity, may also contribute to the pathogenesis of FR. Nerinetide, a peptide that disrupts the interaction of postsynaptic density protein 95 and NMDA receptor (NMDAR) subunit GluN2B, has been shown to increase the chance of favorable outcomes in the AIS subcohort that was treated with EVT but without r-tPA, although no benefit was observed for the overall cohort (patients with AIS treated with EVT with and without r-tPA), which was attributable to the concentration-lowering effect of alteplase on nerinetide [149]. An increased trend toward better neurological recovery was also observed in a phase II trial that compared nelonemdaz, an NMDAR blocker and free radical scavenger, with placebo in patients with anterior circulation LVO who underwent EVT [150]. However, the efficacy of these agents has to be tested in larger RCTs and excitotoxicity as a target of FR has to be elucidated in detail in preclinical studies.

In summary, the incidence of FR after EVT for AIS is high, and the detailed mechanisms remain unclear, which probably include no-reflow, EAR, HT, and poor collateral circulation. Therapeutic strategies targeting these pathophysiological mechanisms have been explored in preclinical research, but translation to the bedside will take time. Since vascular recanalization is not equal to tissue reperfusion, which is a better predictor of outcome, we need to focus more on therapeutic strategies that target tissue reperfusion and attenuate FR.
